# Multimodal Prediction of 3- and 12-Month Outcomes in ICU Patients with Acute Disorders of Consciousness

**DOI:** 10.1007/s12028-023-01816-z

**Published:** 2023-09-11

**Authors:** Moshgan Amiri, Federico Raimondo, Patrick M. Fisher, Melita Cacic Hribljan, Annette Sidaros, Marwan H. Othman, Ivan Zibrandtsen, Ove Bergdal, Maria Louise Fabritius, Adam Espe Hansen, Christian Hassager, Joan Lilja S. Højgaard, Helene Ravnholt Jensen, Niels Vendelbo Knudsen, Emilie Lund Laursen, Jacob E. Møller, Vardan Nersesjan, Miki Nicolic, Sigurdur Thor Sigurdsson, Jacobo D. Sitt, Christine Sølling, Karen Lise Welling, Lisette M. Willumsen, John Hauerberg, Vibeke Andrée Larsen, Martin Ejler Fabricius, Gitte Moos Knudsen, Jesper Kjærgaard, Kirsten Møller, Daniel Kondziella

**Affiliations:** 1 Department of Neurology, Copenhagen University Hospital - Rigshospitalet, Blegdamsvej 9, 2100 Copenhagen, Denmark; 2 Brain and Behaviour, Institute of Neuroscience and Medicine, Research Center Jülich, Jülich, Germany; 3 Institute of Systems Neuroscience, Medical Faculty, Heinrich Heine University Düsseldorf, Düsseldorf, Germany; 4 Neurobiology Research Unit, Copenhagen University Hospital - Rigshospitalet, Copenhagen, Denmark; 5 Department of Drug Design and Pharmacology, University of Copenhagen, Copenhagen, Denmark; 6 Department of Neurophysiology, Copenhagen University Hospital - Rigshospitalet, Copenhagen, Denmark; 7 Department of Neurosurgery, Copenhagen University Hospital - Rigshospitalet, Copenhagen, Denmark; 8 Department of Neuroanaesthesiology, Copenhagen University Hospital - Rigshospitalet, Copenhagen, Denmark; 9 Department of Radiology, Copenhagen University Hospital - Rigshospitalet, Copenhagen, Denmark; 10 Department of Clinical Medicine, University of Copenhagen, Copenhagen, Denmark; 11 Department of Cardiology, Copenhagen University Hospital - Rigshospitalet, Copenhagen, Denmark; 12 Biological and Precision Psychiatry, Copenhagen Research Center for Mental Health, Copenhagen University Hospital, Copenhagen, Denmark; 13 Institut du Cerveau - Paris Brain Institute, Inserm, Centre nationl de la recherche scientifique, Assistance Publique - Hôpitaux de Paris, Sorbonne Université, Hôpital de La Pitié Salpêtrière, Paris, France

**Keywords:** Coma, Consciousness, Functional magnetic resonance imaging, Electroencephalography, Intensive care unit

## Abstract

**Background:**

In intensive care unit (ICU) patients with coma and other disorders of consciousness (DoC), outcome prediction is key to decision-making regarding prognostication, neurorehabilitation, and management of family expectations. Current prediction algorithms are largely based on chronic DoC, whereas multimodal data from acute DoC are scarce. Therefore, the Consciousness in Neurocritical Care Cohort Study Using Electroencephalography and Functional Magnetic Resonance Imaging (i.e. CONNECT-ME; ClinicalTrials.gov identifier: NCT02644265) investigates ICU patients with acute DoC due to traumatic and nontraumatic brain injuries, using electroencephalography (EEG) (resting-state and passive paradigms), functional magnetic resonance imaging (fMRI) (resting-state) and systematic clinical examinations.

**Methods:**

We previously presented results for a subset of patients (*n* = 87) concerning prediction of consciousness levels in the ICU. Now we report 3- and 12-month outcomes in an extended cohort (*n* = 123). Favorable outcome was defined as a modified Rankin Scale score ≤ 3, a cerebral performance category score ≤ 2, and a Glasgow Outcome Scale Extended score ≥ 4. EEG features included visual grading, automated spectral categorization, and support vector machine consciousness classifier. fMRI features included functional connectivity measures from six resting-state networks. Random forest and support vector machine were applied to EEG and fMRI features to predict outcomes. Here, random forest results are presented as areas under the curve (AUC) of receiver operating characteristic curves or accuracy. Cox proportional regression with in-hospital death as a competing risk was used to assess independent clinical predictors of time to favorable outcome.

**Results:**

Between April 2016 and July 2021, we enrolled 123 patients (mean age 51 years, 42% women). Of 82 (66%) ICU survivors, 3- and 12-month outcomes were available for 79 (96%) and 77 (94%), respectively. EEG features predicted both 3-month (AUC 0.79 [95% confidence interval (CI) 0.77–0.82]) and 12-month (AUC 0.74 [95% CI 0.71–0.77]) outcomes. fMRI features appeared to predict 3-month outcome (accuracy 0.69–0.78) both alone and when combined with some EEG features (accuracies 0.73–0.84) but not 12-month outcome (larger sample sizes needed). Independent clinical predictors of time to favorable outcome were younger age (hazard ratio [HR] 1.04 [95% CI 1.02–1.06]), traumatic brain injury (HR 1.94 [95% CI 1.04–3.61]), command-following abilities at admission (HR 2.70 [95% CI 1.40–5.23]), initial brain imaging without severe pathological findings (HR 2.42 [95% CI 1.12–5.22]), improving consciousness in the ICU (HR 5.76 [95% CI 2.41–15.51]), and favorable visual-graded EEG (HR 2.47 [95% CI 1.46–4.19]).

**Conclusions:**

Our results indicate that EEG and fMRI features and readily available clinical data predict short-term outcome of patients with acute DoC and that EEG also predicts 12-month outcome after ICU discharge.

**Supplementary Information:**

The online version contains supplementary material available at 10.1007/s12028-023-01816-z.

## Introduction

Since the first report in 2006 of a patient with cognitive motor dissociation [1], the challenge of identifying patients with brain injury with residual consciousness and predicting their long-term recovery has stimulated a new field of research. This, however, mostly concerns patients with subacute or chronic disorders of consciousness (DoC) in rehabilitation facilities [2, 3].

Each year 2 of 1000 people fall into a coma and are admitted to an intensive care unit (ICU) [4], with the key questions being: Who regains consciousness, and who will make a good functional outcome? Accurate prediction of long-term functional outcomes of patients with acute DoC, including coma, is a major challenge, especially during the early phase in the ICU [5]. Although some DoC survivors enter a state of prolonged unresponsive wakefulness, many recover within weeks to months, and a few patients with DoC may show signs of recovery even years after their brain injury [3, 6]. Accurate prognostication is hence essential for decision-making in the ICU, including decisions about therapeutic management, withdrawal of life-sustaining therapy [7–9], resource allocation and rehabilitation, and management of family expectations. The first step to improve prognostication of patients with acute DoC is accurate determination of their levels of consciousness [10]. This is important because patients with even minimal clinical signs of residual consciousness [11, 12] have more favorable long-term outcomes (as do those with covert consciousness [13–15]). However, determining consciousness levels by routine clinical examinations alone is imprecise [16] because intermittent signs of consciousness are often missed when sensitive systematic ratings scales are omitted [3, 10, 17].

Advanced methods such as functional magnetic resonance imaging (fMRI) and electroencephalography (EEG) can reveal covert signs of consciousness in patients with DoC that are not apparent through clinical examinations [10]. Task-based fMRI and EEG paradigms (i.e., active paradigms) are highly specific in identifying these patients [18, 19], but they may not always accurately detect residual consciousness because of insufficient arousal levels, lack of sustained attention, and fluctuating awareness, which is particularly challenging with patients with acute DoC. Therefore, fMRI and EEG paradigms that involve resting-state (i.e., patients receive no stimulations) or passive paradigms (e.g., addressing the patient by their name or passive eye opening) may be more suitable when assessing patients with acute DoC.

In previous work, we established that resting-state EEG, EEG with external stimulations, and resting-state fMRI can accurately predict consciousness levels in patients with acute DoC during ICU admission [20]. Corroborating our findings, multimodal approaches were recommended in a recent review of neuroimaging-based outcome prediction of patients with DoC [21]. However, prognostication of functional recovery of acute DoC is typically limited to unimodal approaches and certain patient subcategories [6, 14, 19, 22]. Only one study reported 6-month outcome of patients with acute DoC with severe traumatic brain injury (TBI) assessed with both EEG and fMRI [23]. Research reporting the potential of multimodal approaches to predict both early and late functional outcomes of patients with acute DoC in the ICU across a wide range of brain injuries is, to our knowledge, nonexistent.

To bridge this knowledge gap, we investigated whether a multimodal approach consisting of EEG with resting and passive stimulation paradigms, resting-state fMRI, and repeated systematic clinical evaluations could accurately predict functional outcomes of patients with acute DoC with TBI and various nontraumatic brain injuries 3 and 12 months after ICU discharge.

## Methods

**Fig. 1:**
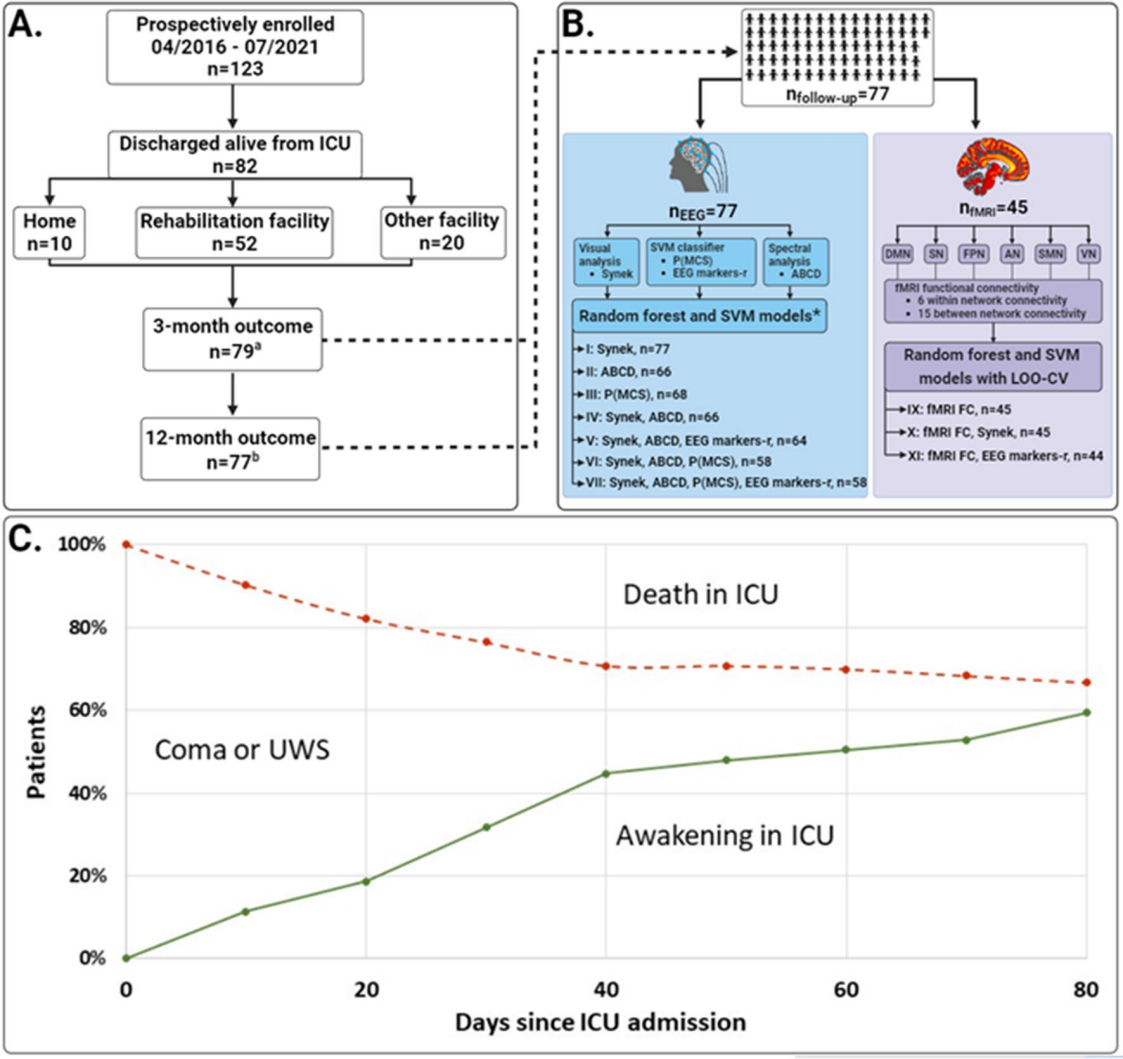
Study flowchart, data assessment strategy and death in ICU. **A.** A total of 123 patients with acute DoC were included, of whom 41 died during ICU admission. Of the 82 patients discharged alive, 10 (12%) patients were discharged directly to their own home, 20 (24%) to other care facilities such as nursing homes, and the remaining 52 (63%) to a high-level neurorehabilitation facility. Three-month follow-up data was available from 79 (96%) patients, and 12-month follow-up data from 77 (94%) patients. **B.** Full sets of 3- and 12-month follow-up data were available for 77 (94%) patients. EEG recordings were available from all patients (blue box), while fMRI resting-state sequences were available from 45 (58%) patients (purple box). EEGs were analyzed with three different approaches; (1) visual manual analysis and scoring according to the Synek scale, (2) automated spectral analysis according to the ABCD model, and (3) a machine learning based SVM consciousness classifier resulting in the probability of being at least in a minimal conscious state (P(MCS)) and 68 EEG markers derived from segments of resting-state EEG (EEG markers-r). Two different machine learning algorithms (i.e., random forest and SVM) were used to conduct seven different predictive models based on EEG features (i.e., models I to VIII) and three different models including fMRI features with or without EEG features (i.e., models IX to XI). Models including fMRI features were assessed with additional LOO-CV procedure due to the limited number of available samples. **C.** This part depicts the proportion of patients in coma or UWS who either awoke to at least MCS- (i.e., regained consciousness to some degree) or died during ICU admission. At time 0 (admission to the ICU) none of the patients were awake (0%) and all were alive (100%). The red line shows the proportion of patients who died in the ICU, and the green line shows the proportion of patients who awoke from coma or UWS in the ICU. During ICU admission, a total of 41 patients (33%) died, while 82 (67%) survived, of whom 73 (59%) awoke prior to ICU discharge. The area between the red and green line indicates the proportion of patients (7%) who remained in coma or UWS at ICU discharge. ^a^Including eight patients who died prior to 3-month follow-up; ^b^Including 13 patients who died prior to 12-month follow-up; * all EEG models were also tested with same-sample data for head-to-head comparison (see also Table 3). Abbreviations: ICU = intensive care unit, EEG = electroencephalography, fMRI = functional magnetic resonance imaging, SVM = support vector machine, DMN = default mode network, SN = salience network, FPN = frontoparietal network, AN =auditory network, SMN = somatosensory network, VN = visual network, LOO-CV = leave-one-out cross-validation, FC = functional connectivity, UWS = unresponsive wakefulness syndrome.

The Consciousness in Neurocritical Care Cohort Study using EEG and fMRI (CONNECT-ME) (ClinicalTrials.gov identifier: NCT02644265) is a tertiary-center prospective, observational diagnostic phase IIb cohort study. Detailed methods of data acquisition and analysis are described in the study protocol [24] and a recent article [20]. Results concerning the prediction of consciousness levels in a subset of patients (*n* = 87) at ICU discharge have been published elsewhere [20]. Here, we evaluated 3- and 12-month functional outcomes in an extended patient cohort (*n* = 123). In the following sections, we provide a brief overview of the methods. Figure 1 shows the flow of patients through the study.

### Patients and Study Design

We prospectively included patients admitted to one of the four ICUs (excluding the neonatal ICU) at Rigshospitalet (Campus Blegdamsvej), Copenhagen University Hospital, Copenhagen, Denmark, between April 2016 and July 2021 and collected demographics, clinical status, and data regarding previous medical history. We included ICU patients aged ≥ 16 years with acute DoC (time from brain injury < 31 days) and Danish- or English-language proficiency who had a clinical indication for structural brain magnetic resonance imaging (MRI) ordered by the treating physician. Clinical examinations, EEG, and fMRI were all performed at the time of enrollment into the study and within a 24-h window or as close to this time window as possible. We aimed for unsedated patients or for the lowest possible sedation levels if patients could not be fully weaned from sedation. As previously described [20], sedation levels were graded as “none or minimal” (i.e., absence of intravenous fentanyl, remifentanil, propofol, midazolam, sodium thiopental, or sevoflurane), “low to moderate” (fentanyl < 500 µg/h or < 200 µg/h combined with propofol, remifentanil < 1,000 µg/h or < 250 µg/h combined with propofol, propofol < 100 mg/h, midazolam < 10 mg/h, sevoflurane < 3%) or “high or very high” (propofol ≥ 100 mg/h, fentanyl ≥ 500 µg/h or ≥ 200 µg/h combined with propofol, remifentanil ≥ 1,000 µg/h or ≥ 250 µg/h combined with propofol, midazolam ≥ 10 mg/h, sevoflurane ≥ 3%, or any dosage of sodium thiopental). Patients with contraindications for MRI, major premorbid neurological deficits (e.g., mental retardation, aphasia, or deafness), and/or acute life-threatening conditions with immediate risk of clinical deterioration were excluded.

### Classification of Consciousness Levels

Briefly, consciousness levels were determined at the time of study enrollment and at ICU discharge using a systematic clinical approach that included sub-elements of the Coma Recovery Scale Revised [17], with the addition of the Glasgow Coma Scale (GCS) [25], and the Full Outline of Unresponsiveness [26]. Furthermore, daily routine neurological examinations were performed by the attending team of physicians, and results were accessed from the electronic health records. We classified patients according to their level of consciousness into the following categories, applying clinical examination techniques as previously described in detail [20]:

- Coma [27, 28]
- Unresponsive wakefulness syndrome (UWS) [29]: only reflex behavior, such as spontaneous eye opening
- Minimally conscious state (MCS − / +) [30, 31]: MCS − , definite signs of nonreflex behavior, such as visual pursuit, localization to noxious stimuli, or relevant emotional response; MCS + , ability to follow simple commands repeatedly but not necessarily consistently
- Emergence from MCS [32]: reliable two-way communication or functional object use
- Locked-in syndrome (LIS) [33]: consistent and reliable communication by rudimentary eye opening

### EEG

Standard 19/25 channel bedside video-EEG (NicoletOne, Natus Medical Inc., Middleton, WI) was recorded with electrodes placed according to the international 10/20 system [34]. All EEGs contained a 10-min resting-state segment, and for reactivity assessment, a segment with stimulations included eye opening; calling the patient by their name; noxious stimuli applied as pressure to the earlobes, fingertips, and sternum; and sensory tactile stimuli applied with a cotton swap to the nostrils.

EEGs were assessed in three different ways, as described previously [20]:

1. Manual visual analysis by two experienced board-certified neurophysiologists (Melita Cacic Hribljan and Annette Sidaros; disagreements resolved by Martin Ejler Fabricius) scoring the EEGs according to the Synek scale [35] (level I to V with increasing level indicating increasing pathology)
2. ABCD spectral analysis by MA and IZ (disagreements resolved by DK) as described by Forgacs et al. [36] (with category A indicating complete corticothalamic disruption and category D indicating full recovery of corticothalamic circuit; segments not clearly falling under A, B, C, or D were classified as “non-ABCD”)
3. A support vector machine (SVM)–based consciousness classifier [37] predicting the probability of the patient’s consciousness level being at least MCS − (P(MCS)) from 68 EEG markers derived separately from EEG resting segments and segments with stimulations.

All investigators performing EEG visual and spectral analyses were unaware of patient outcomes. For other details regarding data cleaning, preprocessing, and specific EEG features included in the aforementioned methods, please refer to our previous publication [20].

### fMRI

A 10-min resting-state scan session with a T2*-weighted echo-planar imaging blood oxygen level–dependent fMRI sequence was performed on 1.5- or 3-Tesla MRI scanners (Siemens, Erlangen, Germany) with 20- or 64-channel head coils, respectively. Preprocessing of fMRI data was performed using SPM12 in MATLAB v2019a (https://www.fil.ion.ucl.ac.uk/spm/software/spm12/) according to our previously described method [20]. Briefly, denoised regional time series were extracted, and region-to-region functional connectivity was estimated by calculating the timewise correlation coefficient (Pearson’s *ρ*) between each pair of regional time series and applying Fisher’s r-to-z transformation to the correlation coefficient. A total of 21 within- and between-network functional connectivity measures were calculated as the average functional connectivity across the set of region-to-region pairs for six resting-state networks (i.e., the default mode network, frontoparietal network, auditory network, salience network, sensorimotor network, and visual network). Investigators assessing fMRI data were unaware of patient outcomes.

### Follow-up Data

We used three outcome scales to assess functional outcome at 3 and 12 months after ICU discharge: (1) the modified Rankin Scale (mRS) [38], (2) the Glasgow Outcome Scale Extended (GOS-E) [39], and (3) the cerebral performance category (CPC) [40] (Box S1). The mRS is used for evaluation of recovery in stroke patients with a focus on the patient’s ability to walk with or without assistance [38]. The GOS-E is an overall functional outcome scale frequently used to collect follow-up data of patients with TBI and includes other aspects of functional recovery, such as the ability to work and socialize and the level of emotional deficits [39]. Finally, the CPC is an evaluation tool to assess the level of recovery of cardiac arrest patients, with regaining of consciousness considered a main aspect [40]. By including all three scales, we aimed at evaluating different aspects of functional recovery, as our study cohort consists of a heterogeneous group of patients regarding the cause of brain injury (i.e., stroke, TBI, cardiac arrest, and other neurological and medical causes). Functional outcome was determined from electronic health records typically based on structural assessments by experienced nursing staff at the high-level rehabilitation facility most surviving patients were discharged to. If sufficient data were not available from health records, patients, family members, or other caregivers were contacted by telephone. Favorable outcome was defined as a combination of an mRS score ≤ 3 (indicating that patients can walk unassisted), a GOS-E score ≥ 4 (indicating that patients can take care of themselves alone for at least 8 h at home), and a CPC score ≤ 2 (indicating that patients are conscious and independent of others for activities of daily living). Patients who died after hospital discharge were included in the primary outcome analysis, whereas patients who died during the ICU stay were excluded, as were patients lost to follow-up.

### Machine Learning Algorithms and Predictive Models

Two machine learning algorithms were used: random forest and SVM. This ensured exploiting both linear and nonlinear interactions. Algorithms were trained to predict binary outcome at 3- and 12-months’ follow-up. Model performance was estimated using stratified fivefold cross-validation (repeated ten times). A special cross-validation scheme (leave-one-out cross-validation [LOO-CV] [41]) was used to evaluate the potential of fMRI features, as the limited fMRI samples available from patients with follow-up outcome did not allow us to obtain reliable estimates with fivefold cross-validation. Algorithm hyperparameters were selected using nested cross-validation and a grid-search procedure. Both unimodal models based on single features (EEG or fMRI features) and multimodal models based on a combination of several features (e.g., combination of EEG and fMRI features) were developed with main outcome measures as binary targets. Furthermore, we conducted a clinical model based on the following clinical characteristics: (1) improving consciousness levels during the ICU stay (i.e., higher level of consciousness at ICU discharge compared with study enrollment), (2) sex, (3) age at admission, (4) preadmission comorbidities, (5) TBI as the cause of injury leading to admission, and (6) command-following abilities at admission. In total, 12 different predictive models (I–XII) were developed and tested with each algorithm based on EEG, fMRI, and clinical features.

Same-sample models were tested for head-to-head comparison of EEG features and clinical features but could not be tested with fMRI features because of the low number of available patients with the full set of EEG features, fMRI features, and outcome measures. Prediction performance of models evaluated with fivefold cross-validation were assessed with area under the curve (AUC) of the receiver operating characteristic curve, sensitivity, and positive predictive value (PPV), whereas performance of the LOO-CV models including fMRI features was assessed with the accuracy measure (ratio of correctly predicted samples over total samples). Pairwise comparison of the AUC of same-sample EEG and clinical models were performed using the corrected *t*-test (two-sided) for comparing machine learning models [42]. *P* values were corrected for multiple comparisons using Bonferroni. AUC, sensitivity, and PPV estimates are reported as mean (95% confidence interval [CI]), and accuracies are reported as a number between 0 and 1. The models hence predict the precision with which favorable outcomes can be distinguished from unfavorable outcomes. All machine learning analyses were done using Julearn and scikit-learn [43].

### Outcome Measures

Our primary target outcome was the binary outcome at 3- and 12-months’ follow-up. Time to favorable outcome was considered a secondary outcome.

### Statistical Analysis

Quantitative data are expressed as mean ± SD or median (interquartile range), and group comparisons were assessed with Student’s *t*-test, the Mann–Whitney *U*-test, or the Kruskal–Wallis test. Categorical data are expressed as numbers (percentages) and were compared using the χ^2^ test or Fisher’s exact test. Cox proportional hazards regression model with in-hospital death considered as a competing risk was used for the assessment of important predictors of time to favorable outcome. Multicollinearity analysis was performed, and variable inflation factor was assessed to avoid high level of correlation between the variables in the regression model. Results are expressed as hazard ratios (HRs) with corresponding 95% CIs and *P* values. The statistical software R version 4.2.0 was used for statistical analysis.

### Data Availability

fMRI data cannot be made fully anonymous and are not publicly available. Other data will be shared upon reasonable request. The code used in the predictive models is available at https://github.com/fraimondo/connectme-followup.

#### Ethics

This study was approved by the Danish Data Protection Agency (RH-2016-191, I-Suite nr:04760) and the Ethics Committee of the Capital Region of Denmark (File-nr.:H-16040845). Written consent was waived because all data were acquired during routine clinical workup. CONNECT-ME is registered with ClinicalTrials.org (identifier: NCT02644265).

## Results

### Demographics and Clinical Characteristics

We included 123 patients (mean age 51 ± 19 years; 51 [42%] women), of whom 82 (67%) were discharged alive from the ICU (Fig. 1 and Table 1). Of the 41 deaths in the ICU, 37 (90%) occurred after withdrawal of life-sustaining therapy. Advanced age, preadmission comorbidity, cardiac arrest as the cause of ICU admission (odds ratio [95% CI] 10.4 [2.46–78.3]), lower GCS motor score at admission, lower total GCS and Full Outline of Unresponsiveness scores at study enrollment, lower consciousness levels at study enrollment, and shorter duration of ICU admission were all significantly associated with death in the ICU (all *P* < 0.05; Table 1). EEG was available from 122 (99%) patients, whereas fMRI was available from 67 (54%) patients. The proportion of patients with fMRI did not differ between those who died in the ICU and patients discharged alive.

**Table 1:** Demographics and clinical characteristics of study population and comparison of patients discharged alive with patients who died in the ICU

Basic characteristics	All (*n* = 123)	Discharged alive from ICU *n* = 82)	Death in ICU *n* = 41)	*P*
Age, years	51 (19)	48 (19)	58 (18)	**0.004**
Female sex	51 (42%)	36 (44%)	15 (37%)	0.447
Prehospital comorbidities^a^				
Any	88 (72%)	54 (66%)	34 (83%)	**0.048**
Cardiopulmonary	46 (37%)	28 (34%)	18 (44%)	0.301
Neurological	30 (24%)	24 (29%)	13 (32%)	0.780
Psychiatric	23 (19%)	17 (21%)	6 (15%)	0.431
Other^b^	60 (49%)	29 (35%)	22 (54%)	0.057
mRS prior to admission	0 (0–1)	0 (0–1)	0 (0–1.5)	0.366
Cause of admission				
Traumatic brain injury	35 (29%)	23 (28%)	12 (29%)	0.883
Ischemic stroke	12 (10%)	6 (7%)	6 (15%)	0.223
Cardiac arrest	11 (9%)	2 (2%)	9 (22%)	**0.001**
Subarachnoid hemorrhage	7 (6%)	7 (9%)	0 (0%)	–
Intracerebral hemorrhage	9 (7%)	7 (9%)	2 (5%)	0.504
Epilepsy	6 (5%)	5 (6%)	1 (2%)	0.428
Other, neurology^c^	20 (16%)	15 (18%)	5 (12%)	0.407
Other, medical/ surgical^d^	23 (19%)	17 (21%)	6 (15%)	0.431
Injury onset to study enrolment, days	15 (14)	17 (15)	11 (11)	0.053
Severe pathology on brain imaging at admission^e^	48 (39%)	31 (38%)	17 (42%)	0.698
fMRI available	67 (54%)	48 (58%)	19 (46%)	0.209
Behavioral scales				
GCS motor score at admission	1 (1–6)	4 (1–6)	1 (1–5)	**0.026**
GCS total score at enrolment	6 (5–9)	7 (6–10)	5 (3–6)	** < 0.001**
FOUR total score at enrolment	8 (6–10)	9 (7–12)	6 (5–8)	** < 0.001**
Command following at admission	48 (39%)	38 (46%)	10 (24%)	**0.031**
Consciousness level at study enrolment				
Coma/UWS	73 (59%)	35 (43%)	38 (93%)	** < 0.001**
MCS−or above	50 (41%)	47 (57%)	3 (7%)	–
Duration of ICU admission, days	31 (26)	35 (28)	22 (16)	**0.006**
Cause of death in ICU, WLST	37 (30%)	–	37 (90%)	–
Discharged from hospital to (*n* = 82)				
Home	10 (8%)	10 (12%)	–	–
Rehabilitation facility	52 (42%)	52 (63%)	–	–
Other care facility	20 (16%)	20 (24%)	–	–
3-month functional outcome			–	–
Favorable	24 (20%)	24 (29%)	–	–
Unfavorable	55 (45%)	55 (67%)	–	–
No data	3 (2%)	3 (4%)	–	–
12-month functional outcome			–	–
Favorable	31 (25%)	31 (38%)	–	–
Unfavorable	46 (37%)	46 (56%)	–	–
No data	5 (4%)	5 (6%)	–	–

Data are presented as *n* (%), mean (SD), and median (interquartile range). Bold values indicate statistical significance

Abbreviations: ICU = intensive care unit, mRS = modified Rankin Scale, fMRI = functional magnetic resonance imaging, GCS = Glasgow Coma Scale, FOUR = Full Outline of Unresponsiveness Score, UWS = unresponsive wakefulness syndrome, MCS = minimally conscious state, WLST = withdrawal of life-sustaing therapy.

^a^Patients could have more than one comorbidity

^b^Other comorbidities included diabetes mellitus, other endocrine, cancer, fibromyalgia, inflammatory bowel disease, MGUS, cirrhosis, osteoporosis, chronic nephropathy, and arthritis

^c^Other causes, neurology included autoimmune encephalitis, brain tumor, hydrocephalus and shunt revision, meningoencephalitis, autoimmune encephalitis, global cerebral oedema, cerebral venous thrombosis, myasthenic crisis, and anoxic ischemic brain damage due to drowning or strangulation

^d^Other causes, medical or surgical included hypoglycemia or hyperglycemia, acute respiratory failure, aortic dissection or ruptured aortic aneurysm, perforated diverticulitis and sepsis, ileus and sepsis, pulmonary embolism, and carbon monoxide poisoning

^e^Severe pathology on brain imaging was defined as Fisher grade ≥ 3 (for subarachnoid hemorrhage), Marshall classification ≥ 3 (for TBI), hemorrhage volume ≥ 30 mL (for intracerebral hemorrhage), strategic brainstem lesions (for ischemic stroke or infratentorial hemorrhage), any visible sign of anoxic brain injury on CT scan (for cardiac arrest), global cortical edema (for patients with brain edema), midline compression, compression of basal cisterns and/or visible signs of hydrocephalus (for patients with any type of brain tumor)

### Functional Outcome and Time to Favorable Outcome

**Fig. 2:**
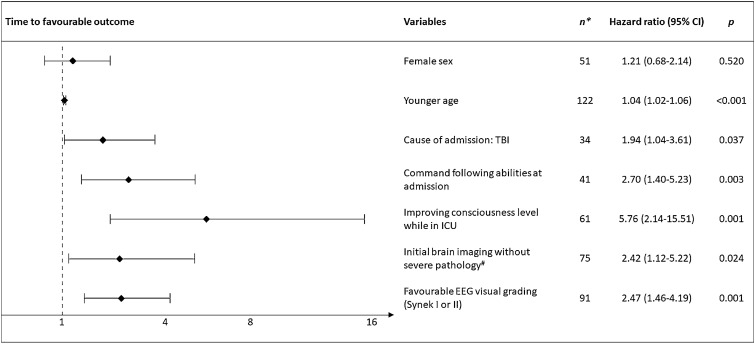
Predictors of time to favorable outcome. This figure depicts independent variables predicting time to favorable outcome (i.e., GOS-E ≥ 4, mRS ≤ 3 and CPC ≤ 2). Death in ICU (*n* = 41) was treated as a competing risk in a multivariate Cox proportional regression model. Younger age, patients with TBI, ability to follow commands at admission, improving consciousness level during ICU, no severe pathological findings at admission brain imaging, and favorable visual grading of EEG (i.e., Synek score I or II) were all independent predictors of earlier recovery. *Of all 123 included patients, one patient without EEG was excluded from this analysis. ^#^Severe pathological findings on brain imaging was defined as Fisher grade ≥ 3 (for subarachnoid hemorrhage), Marshall classification ≥ 3 (for TBI), hemorrhage volume ≥ 30 mL (for intracerebral hemorrhage), strategic hemorrhage or infarct in brainstem (for ischemic stroke or infratentorial hemorrhage), any visible sign of anoxic brain injury on CT scan (for cardiac arrest), global cortical edema (for patients with brain edema), brain tumors with midline compression, compression of basal cisterns and/or signs of hydrocephalus (for patients with any type of brain tumor). Abbreviations: TBI = traumatic brain injury. ICU = intensive care unit, EEG = electroencephalography.

Of the 82 patients discharged alive from the ICU, functional outcome was available from 79 (96%) at 3 months and from 77 (94%) at 12 months (Fig. 1). Thirteen patients (16%) died prior to the 12-month follow-up, of whom eight were dead by the 3-month follow-up. Of the 79 patients with 3-month follow-up data, 26 (33%) had an mRS score of ≤ 3, 24 (30%) had a CPC score of ≤ 2, and 33 (42%) had a GOS-E score of ≥ 4. Of the 77 patients with 12-month follow-up data, 32 (42%) had an mRS score of ≤ 3, 33 (43%) had a CPC score of ≤ 2, and 44 (57%) had a GOS-E score of ≥ 4. Overall, 24 (30%) of the 79 patients had favorable outcome (i.e., favorable functional outcome according to all three outcome scales) at 3 months, and 31 (40%) of 77 had favorable outcome at 12 months. Patients with an unfavorable outcome at both 3 and 12 months were more likely to be discharged from hospital to a high-level rehabilitation facility or another care facility, such as a nursing home, rather than to their own home. Clinical characteristics and comparison of patients with favorable and unfavorable 3- and 12-month functional outcomes are shown in Table 2. As illustrated by Fig. 2, variables independently predicting time to favorable outcome were younger age (HR 1.04 [95% CI 1.02–1.06]), TBI as the cause of ICU admission (HR 1.94 [95% CI 1.04–3.61]), ability to follow commands at admission (HR 2.70 [95% CI 1.40–5.23]), improving consciousness level during the stay in the ICU (HR 5.76 [95% CI 2.14–15.51]), and initial brain imaging without severe pathological findings (HR 2.42 [95% CI 1.12–5.22]). Furthermore, favorable visual EEG grading (i.e., Synek score I or II) (HR 2.47 [95% CI 1.46–4.19]) was also an independent predictor of time to favorable outcome (Fig. 2).

**Table 2:** Comparison of patients with favorable and unfavorable 3- and 12-month functional outcomes

Basic characteristics	3-month outcome (*n* = 79)			12-month outcome (*n* = 77)		
	Favorable (*n* = 24)	Unfavorable (*n* = 55)	*P*	Favorable (*n* = 31)	Unfavorable (*n* = 46)	*P*
Age, years	43 (19)	49 (19)	0.189	38 (16)	54 (18)	** < 0.001**
Female sex	11 (46%)	24 (44%)	0.858	14 (45%)	21 (46%)	0.968
Preadmission comorbidities^a^						
Any	16 (67%)	36 (66%)	0.927	17 (55%)	35 (76%)	0.059
Cardiopulmonary	7 (29%)	19 (35%)	0.658	6 (19%)	20 (44%)	**0.03**
Neurological	3 (13%)	21 (38%)	**0.022**	5 (16%)	19 (42%)	**0.021**
Psychiatric	10 (42%)	7 (13%)	**0.007**	11 (36%)	6 (13%)	**0.026**
Other medical^b^	8 (33%)	20 (36%)	0.809	7 (23%)	21 (46%)	**0.042**
mRS prior to admission	0 (0–0.5)	0 (0–1)	0.098	0 (0–0)	1 (0–2)	**0.005**
Clinical characteristics						
Cause of admission						
Traumatic brain injury	6 (25%)	16 (29%)	0.729	10 (32%)	11 (24%)	0.434
Ischemic stroke	1 (4%)	5 (9%)	0,509	2 (6%)	4 (9%)	0.758
Cardiac arrest	0 (0%)	2 (4%)	1	0 (0%)	2 (4%)	0.513
Subarachnoid hemorrhage	0 (0%)	7 (13%)	0.094	1 (3%)	6 (13%)	0.165
Intracerebral hemorrhage	0 (0%)	7 (13%)	0.094	0 (0%)	7 (15%)	**0.037**
Epilepsy	2 (8%)	3 (5%)	0,644	1 (3%)	4 (9%)	0.395
Other, neurology^c^	8 (33%)	7 (13%)	**0.045**	8 (26%)	7 (15%)	0.27
Other, medical/ surgical^d^	7 (29%)	8 (15%)	0.15	9 (29%)	5 (11%)	0.054
Severe pathology on brain imaging at admission^e^	3 (13%)	28 (51%)	**0.001**	6 (19%)	25 (54%)	**0.002**
fMRI available	11 (46%)	34 (62%)	0.153	18 (58%)	27 (59%)	0.827
Sedation level during fMRI						0.146
None or minimal	4 (36%)	12 (35%)		5 (28%)	11 (41%)	
Low or moderate	4 (36%)	13 (38%)		6 (33%)	11 (41%)	
High or very high	3 (27%)	7 (21%)		7 (39%)	3 (11%)	
Unknown	0 (0%)	2 (6%)		0 (0%)	2 (7%)	
Sedation level during EEG			0.758			0.26
None or minimal	17 (71%)	43 (78%)		21 (68%)	37 (80%)	
Low or moderate	5 (21%)	8 (15%)		6 (19%)	7 (15%)	
High or very high	2 (8%)	4 (7%)		4 (13%)	2 (4%)	
Behavioral scales						
GCS motor score at admission	6 (4–6)	3 (1–6)	**0.013**	5 (1–6)	4 (1–6)	0.5
GCS total score at enrolment	6.5 (5–10)	7 (6–10)	0.492	8 (5–10)	7 (6–9.75)	0.762
FOUR total score at enrolment	8.5 (7–12)	9 (7–12)	0.306	9 (7–12)	8.5 (7–12)	0.243
Command following at admission	18 (75%)	20 (36%)	0.004	18 (58%)	19 (41%)	0.226
Consciousness level at study enrolment			0.878			0.388
Coma/UWS	10 (42%)	24 (44%)	–	11 (36%)	21 (46%)	–
MCS−or above	14 (58%)	31 (56%)	–	20 (65%)	25 (54%)	–
Consciousness level at ICU discharge			0.104			**0.023**
Coma/UWS	1 (4%)	10 (18%)	–	1 (3%)	10 (22%)	–
MCS−or above	23 (96%)	45 (82%)	–	30 (97%)	36 (78%)	–
Duration of ICU admission, days	24 (20)	41 (31)	**0.022**	31 (30)	39 (28)	1.01 (0.99–1.03)
Discharged from hospital to			** < 0.001**			**0.009**
Home	9 (38%)	1 (2%)	–	8 (26%)	1 (2%)	–
Rehabilitation facility	6 (25%)	10 (19%)	–	5 (16%)	11 (24%)	–
Other care facility	9 (38%)	43 (80%)	–	18 (58%)	33 (73%)	–

Data are presented as *n* (%), mean (SD), and median (interquartile range). Bold values indicate statistical significance

Abbreviations: mRS = modified Rankin Scale, fMRI = functional magnetic resonance imaging, EEG = electroencephalography, GCS = Glasgow Coma Scale, FOUR = Full Outline of Unresponsiveness Score, UWS = unresponsive wakefulness syndrome, MCS = minimally conscious state, ICU = intensive care unit.

^a^Patients could have more than one comorbidity

^b^Other comorbidities included diabetes mellitus, other endocrine, cancer, fibromyalgia, inflammatory bowel disease, MGUS, cirrhosis, osteoporosis, chronic nephropathy and arthritis

^c^Other causes, neurology included autoimmune encephalitis, brain tumor, hydrocephalus and shunt revision, meningoencephalitis, autoimmune encephalitis, global cerebral oedema, cerebral venous thrombosis, myasthenic crisis, and anoxic ischemic brain damage due to drowning or strangulation

^d^Other causes, medical or surgical included hypoglycemia or hyperglycemia, acute respiratory failure, aortic dissection or ruptured aortic aneurysm, perforated diverticulitis and sepsis, ileus and sepsis, pulmonary embolism, and carbon monoxide poisoning

^e^Severe pathology on brain imaging was defined as Fisher grade ≥ 3 (for subarachnoid hemorrhage), Marshall classification ≥ 3 (for traumatic brain injury), hemorrhage volume ≥ 30 mL (for intracerebral hemorrhage), strategic brainstem lesions (for ischemic stroke or infratentorial hemorrhage), any visible sign of anoxic brain injury on CT scan (for cardiac arrest), global cortical edema (for patients with brain edema), midline compression, compression of basal cisterns and/or visible signs of hydrocephalus (for patients with any type of brain tumor)

### Machine Learning Predictive Models

In the following sections are results from random forest predictive models, and results from SVM models, statistical analysis of pairwise comparison, and feature importance analysis are presented in the Supplementary Material (Table S1-S2 and Figs. S1-S3).

### EEG Features and Functional Outcome

**Fig. 3:**
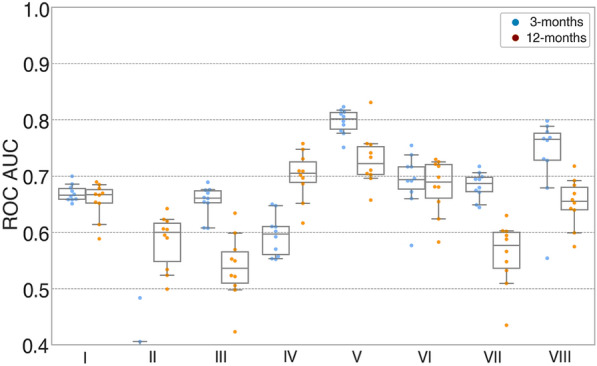
Random forest EEG models with maximum available data predicting 3- and 12-month outcomes. Boxplots illustrating model performances (AUCs) of RF-models based on EEG features predicting 3-month (blue) and 12-month (orange) functional outcomes. Each model is based on the maximum amount of data available (see also Fig. 1). Of the unimodal models (I-III), only model I based on the Synek score could predict both 3- and 12-month outcomes. The highest AUC for predicting both outcomes (AUC_3-month_ 0.79 [0.77–0.82]; AUC_12-month_ 0.74 [0.71–0.77]) were obtained with the combined model (V) based on combination of three EEG features (i.e., Synek score, ABCD categories and EEG markers-r derived from the SVM consciousness classifier). Overall, this figure shows that while Synek score was the only unimodal EEG-model that predicted both 3- and 12-month functional outcomes, all models based on a combination of EEG features (IV–VII) could predict both 3- and 12-month outcomes with AUCs above chance level. A similar pattern was observed for SVM machine learning models (see Fig. S1). Individual EEG random forest models: I = Synek, II = ABCD, III = P(MCS) C. Combined EEG random forest models: IV = Synek + ABCD, V = Synek + ABCD + EEG markers-r, VI = Synek + ABCD + P(MCS), VII = Synek + P(MCS) and VIII = Synek + ABCD + P(MCS) + EEG markers-r

**Fig. 4:**
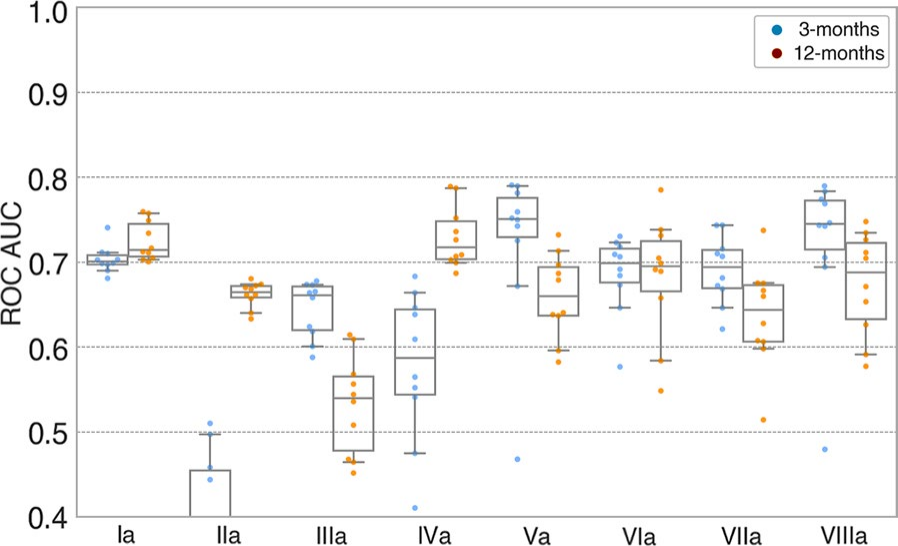
Random forest EEG models with same-sample data predicting 3- and 12-month outcomes. Boxplots illustrating model performances (AUCs) of machine learning models based on EEG features predicting 3-month (blue) and 12-month (orange) functional outcomes. Each model is based on the same samples (n = 58) for head-to-head comparison of EEG features. Of the unimodal models (Ia-IIIa), model Ia based on Synek score outperformed model IIa based on ABCD categories in predicting 3-month outcome (AUC_Synek_ 0.70 [0.69–0.74] vs. AUC_ABCD_ 0.38 [0.31–0.45]). In predicting 12-month outcome, model Ia outperformed model IIIa which was based on P(MCS) measures (AUC_Synek_ 0.70 [0.69–0.74] vs. AUC_P(MCS)_ 0.54 [0.50–0.59]). Of the combined models based on at least three EEG features (Va-VIIa), all models could predict 3- and 12-month outcomes, and none outperformed the others. A similar pattern was observed for SVM machine learning models (see Fig. S2). Individual same-sample EEG random forest models: Ia = Synek, IIa = ABCD, IIIa = P(MCS) C. Combined same-sample EEG random forest models: IVa = Synek + ABCD, Va = Synek + ABCD + EEG markers-r, VIa = Synek + ABCD + P(MCS), VIIa = Synek + P(MCS) and VIIIa = Synek + ABCD + P(MCS) + EEG markers-r. Abbreviations: ROC = receiver operating curve, AUC = area under the curve.

EEG Synek scores were determined for all 77 patients who had available 3- and 12-month outcomes. ABCD scores could be determined for 66 of the 77 patients (the remaining 11 were classified non-ABCD), and P(MCS) could be determined for 68 patients. Sedation levels were high or very high in 6 of the 77 patients (8%) during EEG recording, with no statistically significant effects on functional outcomes (Table 2). Of the predictive models based on individual EEG features (i.e., Synek score, ABCD categories, and P(MCS)), only the Synek score could predict functional outcome at both 3 months (AUC 0.67 [95% CI 0.65–0.70], PPV 0.43 [95% CI 0.34–0.54], sensitivity 0.46 [95% CI 0.41–0.52]) and 12 months (AUC 0.66 [95% CI 0.59–0.69], PPV 0.65 [95% CI 0.57–0.73], sensitivity 0.42 [95% CI 0.41–0.44]). The models based on ABCD categories could not predict 3-month outcome (AUC 0.38 [95% CI 0.34–0.47], PPV 0.13 [95% CI 0.04–0.22], sensitivity 0.24 [95% CI 0.07–0.46]) but could predict 12-month outcome (AUC 0.58 [95% CI 0.50–0.64], PPV 0.61 [95% CI 0.44–0.80], sensitivity 0.21 [95% CI 0.17–0.29]), whereas the models based on P(MCS) could not 12-month outcome (AUC 0.54 [95% CI 0.44–0.63], PPV 0.41 [95% CI 0.31–0.59], sensitivity 0.26 [95% CI 0.18–0.32]) (Table 3 and Fig. 3, models I to III**)**. Head-to-head comparison of the same-sample models based on individual EEG features showed that models based on the Synek score outperformed the ABCD model in predicting 3-month outcome (AUC_Synek_ 0.70 [95% CI 0.68–0.73], AUC_ABCD_ 0.38 [95% CI 0.22–0.51]) and the P(MCS) model in predicting 12-month outcome (AUC_Synek_ 0.72 [95% CI 0.70–0.76], AUC_P(MCS)_ 0.53 [95% CI 0.45–0.61]) (Table 3 and Fig. 4, model Ia compared to model IIa and IIIa).

**Table 3:** Prediction performance of EEG, fMRI, and clinical features in predicting 3- and 12-month functional outcome

Model	Features	N	3-month outcome			12-month outcome		
			AUC	Positive predictive value	Sensitivity	AUC	Positive predictive value	Sensitivity
Random forest EEG models based on all available data								
I	Synek	77	0.67 [0.65–0.70]	0.43 [0.34–0.54]	0.46 [0.41–0.52]	0.66 [0.59–0.69]	0.65 [0.57–0.73]	0.42 [0.41–0.44]
II	ABCD	66	0.38 [0.34–0.47]	0.13 [0.04–0.22]	0.24 [0.07–0.46]	0.58 [0.50–0.64]	0.61 [0.44–0.80]	0.21 [0.17–0.29]
III	P(MCS)	68	0.66 [0.61–0.69]	0.33 [0.25–0.39]	0.64 [0.51–0.76]	0.54 [0.44–0.63]	0.41 [0.31–0.59]	0.26 [0.18–0.32]
IV	Synek, ABCD	66	0.59 [0.55–0.65]	0.45 [0.29–0.59]	0.48 [0.45–0.54]	**0.71 [0.62–0.76]**	0.61 [0.51–0.74]	0.54 [0.44–0.65]
V	Synek, ABCD, EEG markers-r	64	**0.80 [0.76–0.82]**	0.58 [0.48- 0.70]	0.36 [0.29–0.41]	**0.73 [0.67–0.81]**	0.67 [0.58–0.81]	0.54 [0.45–0.63]
VI	Synek, ABCD, P(MCS)	58	0.69 [0.60–0.75]	0.27 [0.024–0.47]	0.34 [0.06–0.46]	0.68 [0.59–0.73]	0.53 [0.42–0.63]	0.37 [0.25–0.46]
VII	Synek, P(MCS)	68	0.68 [0.65–0.71]	0.33 [0.22–0.42]	0.61 [0.41–0.75]	0.56 [0.45–0.62]	0.48 [0.40–0.58]	0.36 [0.29–0.41]
VIII	Synek, ABCD, P(MCS), EEG markers-r	58	**0.74 [0.58–0.80]**	0.38 [0.21–0.50]	0.21 [0.13–0.33]	0.65 [0.58–0.71]	0.51 [0.37–0.74]	0.41 [0.31–0.53]
Random forest EEG models based on same sample data								
Ia	Synek	58	**0.70 [0.68–0.73]**	0.38 [0.27–0.50]	0.53 [0.47–0.59]	**0.72 [0.70–0.76]**	0.52 [0.30–0.71]	0.52 [0.28–0.63]
IIa	ABCD	58	0.38 [0.22–0.51]	0.07 [0.00–0.15]	0.17 [0.02–0.29]	0.66 [0.63–0.68]	0.51 [0.36–0.63]	0.26 [0.23–0.28]
IIIa	P(MCS)	58	0.64 [0.59–0.68]	0.25 [0.12–0.35]	0.49 [0.32–0.69]	0.53 [0.45–0.61]	0.45[0.31–0.62]	0.24 [0.15–0.36]
IVa	Synek, ABCD	58	0.58 [0.43–0.68]	0.29 [0.14–0.47]	0.42 [0.37–0.49]	**0.73 [0.69–0.79]**	0.57 [0.44–0.74]	0.50 [0.35–0.67]
Va	Synek, ABCD, EEG markers-r	58	**0.72 [0.51–0.79]**	0.29 [0.04–0.56]	0.18 [0.01–0.29]	0.66 [0.59–0.73]	0.51 [0.36–0.72]	0.41 [0.28–0.58]
VIa	Synek, ABCD, P(MCS)	58	0.69 [0.59–0.73]	0.26 [0.02–0.46]	0.33 [0.05–0.49]	0.68 [0.56–0.77]	0.54 [0.47–0.74]	0.39 [0.23–0.50]
VIIa	Synek, P(MCS)	58	0.69 [0.62–0.74]	0.28 [0.08–0.40]	0.46 [0.13–0.66]	0.64 [0.53–0.72]	0.56 [0.45–0.73]	0.38 [0.28–0.46]
VIIIa	Synek, ABCD, P(MCS), EEG markers-r	58	**0.72 [0.53–0.79]**	0.33 [0.14–0.51]	0.22 [0.13–0.36]	0.67 [0.58–0.74]	0.52 [0.33–0.68]	0.43 [0.27–0.56]
Random forest fMRI model with LOO-CV procedure								
IX	fMRI FC	45	0.69			0.47		
X	fMRI FC, Synek	45	**0.75**			0.42		
XI	fMRI FC, P(MCS)rest	44	**0.76**			0.45		
Random forest clinical model based on all available data								
XII	Clinical features	77	0.62 [0.55- 0.69]	0.46 [0.27- 0.63]	0.38 [0.25–0.58]	**0.79 [0.77–0.83]**	0.66 [0.63–0.70]	0.73 [0.68–0.77]
Random forest clinical model based on same sample data								
XIIa	Clinical features	58	0.68 [0.60–0.76]	0.22 [0.06–0.41]	0.19 [0.08–0.27]	**0.82 [0.80–0.84]**	0.72 [0.65–0.77]	0.74 [0.64–0.81]

EEG markers-r = 68 EEG markers derived from the EEG resting segments, P(MCS) = support vector machine classifier indicating probability of consciousness derived from EEG markers from the full EEG. P(MCS)_rest_ = support vector machine classifier indicating probability of consciousness derived from EEG markers from the EEG resting segments. Clinical features = age, known preadmission comorbidity, primary injury leading to admission being traumatic brain injury, improving consciousness during intensive care unit, and command following abilities at admission. Numbers in brackets indicate 95% CI. Bold values indicate AUCs or accuracy ≥ 0.70

Abbreviations: AUC = area under the curve, EEG = electroencephalography, fMRI = functional magnetic resonance imaging, LOO-CV = leave-one-out cross-validation. FC = functional connectivity

All but one model (model VII) based on different combinations of EEG features (Fig. 3 and Table 3, models IV, V, VI, VIII and IVa, Va, VIa, VIIIa) could predict functional outcome at both 3- and 12-months’ follow-up. The best combination of AUC, PPV, and sensitivity for prediction of both outcomes was achieved with the model based on the combination of Synek score, ABCD categories, and EEG markers derived from resting EEG segments (3-month outcome: AUC 0.80 [95% CI 0.76–0.82], PPV 0.58 [95% CI 0.48–0.70], sensitivity 0.36 [95% CI 0.29–0.41]; 12-month outcome: AUC 0.73 [95% CI 0.67–0.81], PPV 0.67 [95% CI 0.58–0.81], sensitivity 0.54 [95% CI 0.45–0.63]) (Fig. 3 and Table 3, model V). When comparing the combined EEG same-sample models, all the models performed equally well (Table 3 and Fig. 3, models IVa–VIIIa). Detailed results from the statistical analysis of the pairwise comparison of same-sample EEG models are presented in Table S2. Abbreviations: ROC = receiver operating curve, AUC = area under the curve.

### fMRI Functional Connectivity and Functional Outcome

fMRI features were available for 45 of the 77 patients with both 3- and 12-month outcomes, and 10 of the 45 (22%) received high or very high levels of sedation during the scan, with no statistically significant effects on functional outcomes (Table 2). Because of a limited number of samples with both fMRI data and outcome measures, predictive models including fMRI functional connectivity (FC) were tested with the LOO-CV procedure (Fig. 1, Table 3, and Table S1, models IX-XI). fMRI FC measures tested with both random forest and SVM algorithms showed evidence suggesting that predicting 3-month outcome is possible (random forest model IX: accuracy 0.69; SVM model IX: accuracy 0.78) but not 12-month outcome (random forest model IX: accuracy 0.47; SVM model IX: accuracy 0.47). More samples are required to confirm and correctly estimate the performance of such models.

### Combined EEG and fMRI Features and Functional Outcome

We evaluated prediction of 3- and 12-month functional outcomes with the combination of fMRI FC with the Synek score (*n* = 45) or P(MCS) derived from EEG markers-r (*n* = 44), as depicted in Table 3 (models X and XI). Both combined models showed evidence that predicting 3-month outcome is possible, with accuracies between 0.73 and 0.84, but not 12-month outcome (Table 3 and Table S1, models X and XI), regardless of which algorithm was used.

### Clinical Features and Functional Outcome

The clinical features used to conduct a prediction model were available from all patients with 3- and 12-month outcome data. The clinical model (Table 3, model XII) could predict both 3- and 12-month outcome, with the highest combination of AUC, PPV, and sensitivity achieved for prediction of 12-month outcome (3-month outcome: AUC 0.62 [95% CI 0.55–0.69], PPV 0.46 [95% CI 0.27–0.63], sensitivity 0.38 [95% CI 0.25–0.58]; 12-month outcome: AUC 0.79 [95% CI 0.77–0.83], PPV 0.66 [95% CI 0.63–0.70], sensitivity 0.73 [95% CI 0.68–0.77]). The same-sample model (Table 3, model XIIa) showed the same pattern. When comparing the same-sample clinical model (model XIIa) to EEG models (models Ia–VIIIa), the clinical model seemed to perform equally well to the EEG models for prediction of 3-month outcome and slightly better for prediction of 12-month outcome.

## Discussion

In this, to our knowledge, first prospective multimodal cohort study including 123 ICU patients with acute DoC from various underlying conditions, we show that machine learning algorithms applied to EEG and fMRI features obtained soon after ICU admission can assist in the prediction of 3-month functional outcome, whereas 12-month outcome can only be predicted by EEG features. We also show that the model based on clinical features can predict both outcomes, with highest accuracy for predicting 12-month functional outcome. Thus, we have confirmed readily available independent predictive clinical variables of time to favorable recovery, with the clinical model performing overall as good as EEG models in predicting both outcomes.

EEG features in combination, as well as the EEG Synek score as an individual model, predicted both 3- and 12-month functional outcomes (Fig. 3 and Table 3), whereas all models based on fMRI FC measures could only predict 3-month outcome (Table 3). EEG recordings were available from all 77 patients with outcome measures at both 3 and 12 months, whereas we only had fMRI sequences from 45 of these patients, thus resulting in a substantially reduced amount of data available for the fMRI feature models. Although the quality of the data that underly machine learning models is crucial, data quantity is also important because data sets with many variables but limited number of samples introduce high level of variance, rendering the models imbalanced [44]. Despite our relatively large population of patients with acute DoC, our results, especially those including fMRI features, should therefore be interpreted with caution until further validation from ongoing multicenter studies [45]. These factors may also explain the relatively low PPV and sensitivities despite high AUCs of the combined EEG models, which were based on data from patients with a complete data set including all EEG features (*n* = 58). High levels of sedation can have a significant impact on resting EEG measures and may affect the accuracy of EEG models used for prediction. However, in our cohort, only six patients (8%) with both 3- and 12-month outcome measures available were under high levels of sedation during their EEG recordings, and therefore we do not consider sedation a significant factor affecting our results. However, 10 of 45 patients (22%) with fMRI sequences received high levels of sedation during their scans, which may have had an impact on the data, but we did not find any statistically significant differences in sedation levels when comparing patients with favorable and unfavorable outcomes.

Despite the aforementioned limitations, we could show that most EEG features predicted both early and late functional outcomes individually and in various combinations (Fig. 3 and Table 3). This is an important finding because EEG is much more available bedside in the ICU than advanced neuroimaging, such as fMRI, and EEG features like ours can be easily implemented in an ICU setting.

When comparing the individual EEG features head-to-head with the same-sample models (Fig. 4 and Table 3), we found that the Synek score outperformed the ABCD categories for the prediction of short-term outcome and the SVM classifier derived P(MCS) for the prediction of long-term outcome. This finding may be explained by the fact that the Synek score was assessed manually by two board-certified electroencephalographers with many years of experience with ICU EEG, whereas the ABCD and P(MCS) features were initially developed in more homogenous patient groups (i.e., homogenous cardiac arrest [33] and chronic DoC cohorts [36, 37] vs. acute DoC cohort with heterogeneous brain injuries) than ours. Furthermore, visual analysis of EEGs is routinely used for prognostication in ICU populations like the present cohort, which may also explain the higher performance of the models based on the Synek score. Still, we could show that combining different EEG features resulted in the best predictive performance of the models, regardless of the algorithm used (Table 3 and Table S1). These are important findings because most ICU sites with patients with acute DoC do not have the resources to perform advanced EEG assessment using machine learning classifiers. These sites can thus safely rely on experienced electroencephalographers using established criteria for visual EEG analyses instead. If the necessary electroencephalographer expertise is unavailable, however, external data-driven analysis of EEGs may become a suitable option for those sites in the near future.

We also show that EEG models are overall comparable to a model based on clinical features for prediction of 3-month outcome, while performing slightly worse for prediction of 12-month outcome. It is not surprising that the clinical model performs well in predicting especially long-term outcome of this patient group when considering that readily available clinical features play a significant role in end-of-life decision-making in the ICU. Thus, the patients who survive in the ICU are a selected group of patients expected to perform better based at least partially on their clinical characteristics than those who died in the ICU, where most deaths were due to expected poor prognosis and thus withdrawal of life-sustaining therapy (Table 1).

Models including fMRI features were tested with a LOO-CV procedure because of the limited number of available samples. Results indicate that fMRI FC both alone and in combination with some EEG features may be useful to predict early functional outcome at 3 months (Table 3) but not (yet) late outcome at 12 months. The LOO-CV procedure limits data waste and is therefore primarily used for small data sets, but a major limitation is that the results are prone to optimistic interpretation and therefore need external validation in larger data sets [41].

In the first article from CONNECT-ME [20], we found that EEG and fMRI features predicted levels of consciousness of patients with acute DoC at the time of ICU discharge. Importantly, EEG and fMRI were performed without active consciousness paradigms; thus, patients likely had different degrees of residual consciousness (e.g., including those who could not have participated in active paradigms [10]). Collectively, our findings indicate that both EEG and fMRI have the potential not only to predict level of consciousness during ICU admission [20] but also to predict functional outcome of patients with brain injury of various causes resulting in acute DoC in the early phase of hospitalization and (EEG, at least) up to 1 year after discharge from the ICU.

In line with a recent study about recovery trajectories of patients with cognitive motor dissociation [14], we additionally identified readily available clinical features as independent predictors of time to favorable functional outcome (Fig. 2). In our heterogenous patient cohort reflecting a real-life ICU setting, we confirmed that TBI is related to earlier recovery. Furthermore, patients who were younger, could follow commands at ICU admission, had no severe pathological findings on initial brain imaging, and showed improving consciousness level in the ICU also recovered earlier. Similarly, patients with favorable functional outcomes at 3 and 12 months were more likely to be discharged directly to their own home, whereas patients with unfavorable outcome were more often discharged to rehabilitation facilities and nursing homes (Table 2). This is explained by the fact that patients with more severe injuries needed a higher level of care and were thus discharged to facilities with a higher level of rehabilitation resources. All these findings can help clinicians when guiding patient families about the prospects of recovery, including the time it takes to achieve a good recovery.

Several limitations need to be considered. As a single-center study, CONNECT-ME is susceptible to sampling bias. Our follow-up data were primarily collected through electronic health records based on notes from trained nursing staff who routinely collect functional outcome data from ICU patients, especially those discharged to high facility rehabilitation centers. Because most of the follow-up data were not collected firsthand by the research team, we acknowledge there is a risk of bias. Taking this into consideration, we chose a composite binary outcome measure (i.e., favorable vs. unfavorable) instead of an in-depth analysis of the respective outcome scales.

A relatively large number of patients (33%) died in the ICU, most because of withdrawal of life-sustaining therapy because of a presumed poor prognosis. Although the current study included 123 patients, data from only 77 patients were available for the final analysis of 12-month outcomes. Thus, the remaining cohort with available follow-up data consisted of patients who were expected to regain better functional outcome. This skewed the data set used in the machine learning models. The predictive performance of these models may hence have been biased in that they lacked the (potential) clinical trajectories of patients who had life-sustaining therapy withdrawn. To account for this bias to some extent, in our analysis of independent variables related to time to favorable outcome, we included in-hospital death as a competing risk in the multivariate Cox proportional hazards regression model. Still, death due to withdrawal of life-sustaining therapy in the ICU remains an important limitation and cannot be fully accounted for when studying ICU patients with acute severe brain injury and DoC. Furthermore, the heterogeneity of the brain injuries studied made subgroup analysis other than TBI vs. non-TBI impractical because of the small numbers in each subgroup.

Because EEG is more available in the ICU than fMRI, it is routinely used for prognostication of patients with acute DoC, especially of those admitted post cardiac arrest [46]. Excluding patients who died in the ICU may therefore have decreased the performance of the EEG models as well. Additionally, of the three methods used for EEG analysis, the visual scoring and ABCD scale are subjective and may introduce bias even though the investigators analyzing EEG in our study were blinded to outcomes.

MRI scans are logistically very challenging to obtain in the ICU and are thus less often performed than EEG, which might be yet another selection bias, affecting the fMRI models owing to exclusion of patients without available fMRI. However, in our cohort, we found no statistically significant difference in the frequency with which fMRI was performed when comparing patients who died in the ICU with those who were discharged alive (Table 1) or when comparing patients with favorable outcome with those with unfavorable outcome (Table 2), suggesting this might be of lesser importance to the overall results. Our study population is a heterogeneous group of patients with various causes of DoC, rendering subgroup analysis unreliable because of the low number of patients in each group. Thus, further validation is needed to confirm our findings.

On the positive side, our findings are generalizable to a real-life ICU setting and patients with acute DoC with various causes of brain injury. We also evaluated functional outcome in our cohort by using three different outcome scales designed for stroke (mRS) [38], TBI (GOS-E) [47], and cardiac arrest (CPC) [40] patients to account for the heterogenicity of our patients. Owing to logistical challenges and resources needed for advanced data analyses, to our knowledge, no previous EEG/fMRI study has managed to investigate acute DoC in a larger ICU cohort or with a longer follow-up than ours.

## Conclusions

We show that EEG early during ICU admission predicted both 3- and 12-month functional outcomes of patients with acute DoC with various causes of brain injury and that fMRI resting-state measures might be useful to predict 3-month outcome. Furthermore, young age, TBI, initial brain imaging without severe pathological findings, ability to follow commands during ICU admission, improving consciousness level during the ICU stay, and favorable visual EEG grading all independently predicted shorter time to favorable functional outcome. In summary, we suggest that combining EEG- and fMRI-based machine learning models with readily available clinical data allows for short-term outcome prediction of patients with coma and other acute DoC and potentially can predict long-term outcome up to 1 year after ICU discharge.

### Supplementary Information

Below is the link to the electronic supplementary material.

Supplementary file1 (DOCX 828 KB)
