# Supplementary material for: Multimodal Prediction of 3- and 12-Month Outcomes in ICU Patients with Acute Disorders of Consciousness
Source: Neurocrit Care. 2023 Sep 11;40(2):718–33. doi: 10.1007/s12028-023-01816-z (PMC10959792; doi:10.1007/s12028-023-01816-z)

**Supplementary data**

1. **Box S1:** Functional outcome scales
2. **Table S1:** Prediction performance of SVM models predicting 3- and 12-month functional follow-up
3. **Table S2:** Statistical test with pairwise comparison of same sample EEG models
4. **Figure S1:** Boxplots of SVM unimodal and multimodal models based on maximum available data
5. **Figure S2:** Boxplots of SVM same-sample models based on full-set data
6. **Figure S3:** SHAP analysis of EEG model VIII based on all EEG features

| **Box S1. Functional outcome scales** | | |
| --- | --- | --- |
| **Scale** | **Categories/levels** | **Explanation** |
| **Modified Rankin scale (mRS)** | 0: No current symptoms | - |
|  | 1: No significant disability | Able to carry out all usual activities despite minor symptoms |
|  | 2: Slight disability | Unable to carry out all usual activities, but able to look after daily affairs without help from others |
|  | 3: Moderate disability | Require some help but can walk unassisted |
|  | 4: Moderately severe disability | Assistance needed to attend bodily needs. Unable to walk unassisted |
|  | 5: Severe disability | Constant care needed. Patient is bedridden |
|  | 6: Dead | - |
|  | | |
| **Glasgow Outcome Scale Extended**  **(GOS-E)** | 1: Dead | - |
|  | 2: Persistent vegetative state | Only reflex behavior, inability to communicate |
|  | 3: Severe disability (lower) | Requires frequent help from other most of the time daily |
|  | 4: Severe disability (upper) | Can take of oneself alone for minimum 8 hours per day |
|  | 5: Moderate disability (lower) | Able to work in sheltered workshop or not at all |
|  | 6: Moderate disability (upper) | Work capacity reduced. Social activities reduced to less than 50% of preinjury status |
|  | 7: Good recovery (lower) | Minor issues effecting daily living |
|  | 8: Good recovery (upper) | No current problems effecting daily living |
|  | | |
| **Cerebral Performance Category (CPC)** | 1: Good cerebral performance | Conscious, alert and able to work despite minor deficits. |
|  | 2: Moderate cerebral disability | Conscious, independent of other for daily life activities. Can work in sheltered environment |
|  | 3: Severe cerebral disability | Conscious, dependent on other for daily support. Can range from ambulatory to severe dementia/paralysis |
|  | 4: Coma or vegetative state | Unaware without any ability to interact with the environment |
|  | 5: Dead/Brain dead | - |
| Components of the different functional outcome scales. The green lines show threshold between favorable and unfavorable outcome for each scale | | |

| **Table S1** Prediction performance of EEG and fMRI-features in predicting 3- and 12-month functional outcome | | | | | | | | | | | | |
| --- | --- | --- | --- | --- | --- | --- | --- | --- | --- | --- | --- | --- |
| **Support Vector Machine EEG-models based on all available data** | | | | | | | | | | | | |
|  | | | **3-month** | | | | | **12-month** | | | | |
| **Model** | **Features** | **N** | **AUC** | | **Positive predictive value** | | **Sensitivity** | **AUC** | | **Positive predictive value** | | **Sensitivity** |
| **I** | Synek | 77 | 0.67 [0.65-0.69] | | 0.28 [0.13-0.39] | | 0.55 [0.34-0.77] | 0.56 [0.47-0.59] | | 0.25 [0.12-0.38] | | 0.54 [0.25-0.73] |
| **II** | ABCD | 66 | 0.39 [0.33-0.44] | | 0.19 [0.12-0.29] | | 0.61 [0.4- 0.96] | 0.62 [0.60-0.64] | | 0.65 [0.46-0.83] | | 0.23 [0.21-0.29] |
| **III** | P(MCS) | 68 | **0.70 [0.67-0.73]** | | 0.14 [0.01-0.25] | | 0.33 [0.05-0.62] | 0.61 [0.57-0.67] | | 0.24 [0.10-0.34] | | 0.41 [0.22-0.63] |
| **IV** | Synek, ABCD | 66 | 0.63 [0.55-0.69] | | 0.30 [0.18-0.44] | | 0.53 [0.26-0.91] | 0.61 [0.54-0.66] | | 0.49 [0.17-0.64] | | 0.33 [0.16-0.44] |
| **V** | Synek, ABCD, EEG markers-r | 64 | **0.74 [0.66-0.77]** | | 0.45 [0.30-0.56] | | 0.50 [0.33-0.70] | 0.68 [0.60-0.75] | | 0.56 [0.46-0.68] | | 0.55 [0.41-0.71] |
| **VI** | Synek, ABCD, P(MCS) | 58 | **0.79 [0.78-0.81]** | | 0.31 [0.12-0.42] | | 0.44 [0.23-0.59] | 0.68 [0.61-0.75] | | 0.40 [0.29-0.53] | | 0.38 [0.21-0.52] |
| **VII** | Synek, P(MCS) | 68 | 0.69 [0.67-0.72] | | 0.15 [0.06-0.28] | | 0.38 [0.16-0.53] | 0.57 [0.51-0.62] | | 0.19 [0.11-0.31] | | 0.37 [0.10-0.60] |
| **VIII** | Synek, ABCD, P(MCS), EEG markers-r | 58 | 0.67 [0.58-0.75] | | 0.29 [0.10-0.45] | | 0.37 [0.19-0.59] | 0.64 [0.51-0.74] | | 0.45 [0.28-0.59] | | 0.45 [0.31-0.55] |
| **Support Vector Machine same sample EEG-models** | | | | | | | | | | | | |
| **Ia** | Synek | 58 | **0.70 [0.68-0.73]** | | 0.24 [0.08-0.42] | | 0.35 [0.13-0.56] | 0.47 [0.28-0.58] | | 0.30 [0.14-0.47] | | 0.51 [0.35-0.69] |
| **IIa** | ABCD | 58 | 0.43 [0.35-0.53] | | 0.023 [0.0-0.10] | | 0.03 [0.0-0.12] | 0.67 [0.65-0.69] | | 0.61 [0.48-0.80] | | 0.30 [0.28-0.38] |
| **IIIa** | P(MCS) | 58 | **0.72 [0.68-0.78]** | | 0.25 [0.01-0.43] | | 0.47 [0.23-0.65] | 0.63 [0.50-0.71] | | 0.40 [0.23-0.57] | | 0.56 [0.37-0.74] |
| **IVa** | Synek, ABCD | 58 | 0.64 [0.55-0.72] | | 0.20 [0.06-0.37] | | 0.29 [0.08-0.48] | 0.66 [0.57-0.73] | | 0.51 [0.36-0.62] | | 0.30 [0.19-0.53] |
| **Va** | Synek, ABCD, EEG markers-r | 58 | 0.67 [0.58-0.77] | | 0.27 [0.10-0.41] | | 0.33 [0.18-0.49] | 0.64 [0.52-0.74] | | 0.45 [0.29-0.55] | | 0.46 [0.35-0.60] |
| **VIa** | Synek, ABCD, P(MCS) | 58 | 0.69 [0.63-0.78] | | 0.31 [0.12-0.42] | | 0.44 [0.23-0.59] | 0.68 [0.61-0.75] | | 0.40 [0.29-0.53] | | 0.38 [0.21-0.52] |
| **VIIa** | Synek, P(MCS) | 58 | **0.75 [0.72-0.81]** | | 0.30 [0.14-0.43] | | 0.49 [0.27-0.68] | 0.56 [0.41-0.68] | | 0.25 [0.13-0.39] | | 0.39 [0.26-0.51] |
| **VIIIa** | Synek, ABCD, P(MCS), EEG markers-r | 58 | 0.67 [0.58-0.75] | | 0.29 [0.10-0.45] | | 0.37 [0.19-0.59] | 0.64 [0.51-0.74] | | 0.45 [0.28-0.59] | | 0.45 [0.31-0.55] |
| **Support Vector Machine fMRI-model with LOO-CV procedure** | | | | | | | | | | | | |
|  |  |  | **Accuracy: 3-month outcome** | | | | | **Accuracy: 12-month outcome** | | | | |
| **IX** | fMRI FC | 45 | **0.78** | | | | | 0.47 | | | | |
| **X** | fMRI FC, Synek | 45 | **0.73** | | | | | 0.42 | | | | |
| **XI** | fMRI FC, P(MCS)rest | 44 | **0.84** | | | | | 0.61 | | | | |
| **Support Vector Machine clinical model based on all available data** | | | | | | | | | | | | |
|  |  |  | **3-month** | | | | | **12-month** | | | | |
| **XII** | Clinical features | 77 | 0.66 [0.62-0.71] | 0.31 [0.18-0.45] | | 0.60 [0.42-0.74] | | **0.78 [0.74-0.80]** | 0.62 [0.57-0.67] | | 0.77 [0.71-0.83] | |
| **Support Vector Machine clinical model based on same sample data** | | | | | | | | | | | | |
| **XIIa** | Clinical features | 58 | 0.57 [0.49-0.65] | 0.10 [0.01-0.21] | | 0.21 [0.03-0.42] | | **0.82 [0.80-0.87]** | 0.67 [0.56-0.74] | | 0.78 [0.73-0.82] | |
| EEG markers-r = 68 EEG markers derived from the EEG resting segments, P(MCS) = Support Vector Machine classifier indicating probability of consciousness derived from EEG markers from the full EEG, LOO-CV = Leave-One-Out Cross-Validation, FC = functional connectivity, P(MCS)_rest_ = Support Vector Machine classifier indicating probability of consciousness derived from EEG markers from the EEG resting segments. Numbers in brackets indicate 95% CI. **Bold** values indicate AUCs or accuracy ≥ 0.70 | | | | | | | | | | | | |

| **Table S2** Statistical test for pairwise comparison of ROC AUC scores across same sample EEG models (Model 1 versus Model 2) | | | | | | | |
| --- | --- | --- | --- | --- | --- | --- | --- |
| **Comparison of Random-Forest models** | | | | | | | |
| **Models** | | **3 months outcome** | | | **12 months outcome** | | |
| **Model 1** | **Model 2** | **t-stat** | ***p*** | **Corrected *p*** | **t-stat** | ***p*** | **Corrected *p*** |
| Synek | ABCD | 2.89E+15 | **0.01** | 0.16 | 0.66 | 0.51 | 1.0 |
| Synek | P(MCS) | 0.77 | 0.44 | 1.0 | 1.90E+15 | 0.06 | 1.0 |
| Synek | Synek, ABCD | 1.50E+15 | 0.14 | 1.0 | -0.07 | 0.94 | 1.0 |
| Synek | Synek, P(MCS) | 0.17 | 0.87 | 1.0 | 0.84 | 0.41 | 1.0 |
| Synek | Synek, ABCD, EEG markers-r | -0.26 | 0.80 | 1.0 | 0.63 | 0.53 | 1.0 |
| Synek | Synek, ABCD, P(MCS) | 0.23 | 0.82 | 1.0 | 0.49 | 0.63 | 1.0 |
| Synek | Synek, ABCD, P(MCS), EEG markers- | -0.23 | 0.82 | 1.0 | 0.48 | 0.63 | 1.0 |
| ABCD | P(MCS) | -1.96E+16 | 0.06 | 1.0 | -1.30E+16 | 0.20 | 1.0 |
| ABCD | Synek, ABCD | -2.69E+15 | **0.01** | 0.27 | -1.07E+16 | 0.29 | 1.0 |
| ABCD | Synek, P(MCS) | -2.14E+15 | **0.04** | 1.0 | -0.21 | 0.83 | 1.0 |
| ABCD | Synek, ABCD, EEG markers-r | 2.51E+16 | **0.02** | 0.43 | -0.015 | 0.99 | 1.0 |
| ABCD | Synek, ABCD, P(MCS) | -2.23E+16 | **0.03** | 0.85 | -0.24 | 0.81 | 1.0 |
| ABCD | Synek, ABCD, P(MCS), EEG markers- | -2.61E+16 | **0.01** | 0.34 | 0.12 | 0.90 | 1.0 |
| P(MCS) | Synek, ABCD | -0.58 | 0.56 | 1.0 | -1.98E+16 | 0.05 | 1.0 |
| P(MCS) | Synek, P(MCS) | 0.83 | 0.41 | 1.0 | 1.93E+16 | 0.06 | 1.0 |
| P(MCS) | Synek, ABCD, EEG markers-r | 0.76 | 0.45 | 1.0 | 1.17E+16 | 0.25 | 1.0 |
| P(MCS) | Synek, ABCD, P(MCS) | 0.77 | 0.45 | 1.0 | -2.44E+16 | **0.02** | 0.51 |
| P(MCS) | Synek, ABCD, P(MCS), EEG markers- | 0.66 | 0.51 | 1.0 | 1.37E+16 | 0.18 | 1.0 |
| Synek, ABCD | Synek, P(MCS) | -0.89 | 0.38 | 1.0 | -0.88 | 0.38 | 1.0 |
| Synek, ABCD | Synek, ABCD, EEG markers-r | 1.19E+16 | 0.24 | 1.0 | -0.68 | 0.50 | 1.0 |
| Synek, ABCD | Synek, ABCD, P(MCS) | -0.90 | 0.37 | 1.0 | 0.57 | 0.57 | 1.0 |
| Synek, ABCD | Synek, ABCD, P(MCS), EEG markers- | -1.19E+16 | 0.23 | 1.0 | -0.55 | 0.58 | 1.0 |
| Synek, P(MCS) | Synek, ABCD, EEG markers-r | 0.32 | 0.75 | 1.0 | -0.21 | 0.83 | 1.0 |
| Synek, P(MCS) | Synek, ABCD, P(MCS) | -0.13 | 0.90 | 1.0 | -0.60 | 0.55 | 1.0 |
| Synek, P(MCS) | Synek, ABCD, P(MCS), EEG markers- | 0.28 | 0.78 | 1.0 | 0.37 | 0.71 | 1.0 |
| Synek, ABCD, EEG markers-r | Synek, ABCD, P(MCS) | 0.38 | 0.71 | 1.0 | -0.24 | 0.81 | 1.0 |
| Synek, ABCD, EEG markers-r | Synek, ABCD, P(MCS), EEG markers- | 0.01 | 1.0 | 1.0 | 0.38 | 0.70 | 1.0 |
| Synek, ABCD, P(MCS) | Synek, ABCD, P(MCS), EEG markers-r | 0.33 | 0.75 | 1.0 | -0.09 | 0.93 | 1.0 |
| **Comparison of Support Vector Machine models** | | | | | | | |
| Synek | ABCD | -2.31E+16 | **0.03** | 0.71 | -1.44E+16 | 0.16 | 1.0 |
| Synek | P(MCS) | 0.34 | 0.74 | 1.0 | -1.59E+16 | 0.12 | 1.0 |
| Synek | Synek, ABCD | -1.05E+16 | 0.30 | 1.0 | 1.52E+16 | 0.14 | 1.0 |
| Synek | Synek, P(MCS) | 0.92 | 0.36 | 1.0 | -1.07E+16 | 0.29 | 1.0 |
| Synek | Synek, ABCD, EEG markers-r | -0.53 | 0.60 | 1.0 | -1.45E+16 | 0.15 | 1.0 |
| Synek | Synek, ABCD, P(MCS) | -0.12 | 0.90 | 1.0 | 1.84E+16 | 0.07 | 1.0 |
| Synek | Synek, ABCD, P(MCS), EEG markers- | 0.46 | 0.65 | 1.0 | 1.50E+16 | 0.14 | 1.0 |
| ABCD | P(MCS) | -2.39E+15 | **0.02** | 0.58 | -0.29 | 0.78 | 1.0 |
| ABCD | Synek, ABCD | -2.37E+15 | **0.02** | 0.60 | -0.15 | 0.88 | 1.0 |
| ABCD | Synek, P(MCS) | 2.41E+15 | **0.02** | 0.55 | -0.85 | 0.40 | 1.0 |
| ABCD | Synek, ABCD, EEG markers-r | 2.14E+15 | **0.04** | 1.0 | -0.28 | 0.78 | 1.0 |
| ABCD | Synek, ABCD, P(MCS) | -2.31E+15 | **0.02** | 0.70 | 0.24 | 0.81 | 1.0 |
| ABCD | Synek, ABCD, P(MCS), EEG markers- | -2.13E+16 | **0.04** | 1.0 | -0.24 | 0.81 | 1.0 |
| P(MCS) | Synek, ABCD | 1.05E+16 | 0.30 | 1.0 | 0.25 | 0.80 | 1.0 |
| P(MCS) | Synek, P(MCS) | 0.57 | 0.57 | 1.0 | 1.31E+16 | 0.19 | 1.0 |
| P(MCS) | Synek, ABCD, EEG markers-r | -0.60 | 0.55 | 1.0 | -0.08 | 0.94 | 1.0 |
| P(MCS) | Synek, ABCD, P(MCS) | 0.41 | 0.69 | 1.0 | 0.45 | 0.64 | 1.0 |
| P(MCS) | Synek, ABCD, P(MCS), EEG markers- | 0.56 | 0.58 | 1.0 | 0.11 | 0.92 | 1.0 |
| Synek, ABCD | Synek, P(MCS) | 1.37E+16 | 0.18 | 1.0 | 0.87 | 0.39 | 1.0 |
| Synek, ABCD | Synek, ABCD, EEG markers-r | 0.29 | 0.77 | 1.0 | 0.19 | 0.85 | 1.0 |
| Synek, ABCD | Synek, ABCD, P(MCS) | -0.71 | 0.48 | 1.0 | 0.34 | 0.73 | 1.0 |
| Synek, ABCD | Synek, ABCD, P(MCS), EEG markers- | -0.30 | 0.76 | 1.0 | -0.15 | 0.88 | 1.0 |
| Synek, P(MCS) | Synek, ABCD, EEG markers-r | -0.96 | 0.34 | 1.0 | -0.70 | 0.49 | 1.0 |
| Synek, P(MCS) | Synek, ABCD, P(MCS) | 0.93 | 0.35 | 1.0 | 1.18E+16 | 0.24 | 1.0 |
| Synek, P(MCS) | Synek, ABCD, P(MCS), EEG markers- | 0.93 | 0.36 | 1.0 | 0.75 | 0.46 | 1.0 |
| Synek, ABCD, EEG markers-r | Synek, ABCD, P(MCS) | -0.29 | 0.77 | 1.0 | 0.41 | 0.69 | 1.0 |
| Synek, ABCD, EEG markers-r | Synek, ABCD, P(MCS), EEG markers- | -0.09 | 0.93 | 1.0 | 0.12 | 0.91 | 1.0 |
| Synek, ABCD, P(MCS) | Synek, ABCD, P(MCS), EEG markers-r | 0.27 | 0.79 | 1.0 | 0.37 | 0.72 | 1.0 |
| EEG markers-r = 68 EEG markers derived from the EEG resting segments, P(MCS) = Support Vector Machine classifier indicating probability of consciousness derived from EEG markers from the full EEG. Bold values indicate *p*<0.05. | | | | | | | |

**Figure S1. Support Vector Machine EEG models with maximum available data predicting 3- and 12-month outcomes.** Boxplots illustrating model performances (AUCs) of SVM-models based on EEG-features predicting 3-month (**blue**) and 12-month (**orange**) functional outcomes. Each model is based on the maximum amount of data available (see also **Fig. 1**). Of the unimodal models (**I-III**), both model **I** and **III** based on the Synek score and P(MCS), respectively, could predict both 3- and 12-month outcome. Of the combined models (Models **IV-VIII**) the highest AUC for predicting both outcomes were obtained with models **V-VI** based on all three EEG-features (i.e., Synek score, ABCD categories, P(MCS) or EEG markers-r). In sum, this figure shows that while not all unimodal EEG models could predict both 3- and 12-month functional outcomes, all models based on a combination of the three EEG-features (**IV-VIII**) could predict both 3- and 12-month outcomes with AUCs above chance level. *Individual EEG SVM-models:* **I**=Synek, **II**=ABCD, **III**=P(MCS). *Combined EEG SVM-models*: **IV**=Synek + ABCD, **V**=Synek + ABCD + EEG markers-r, **VI**= Synek + ABCD + P(MCS), **VII**= Synek + P(MCS) and **VIII**= Synek + ABCD + P(MCS) + EEG markers-r


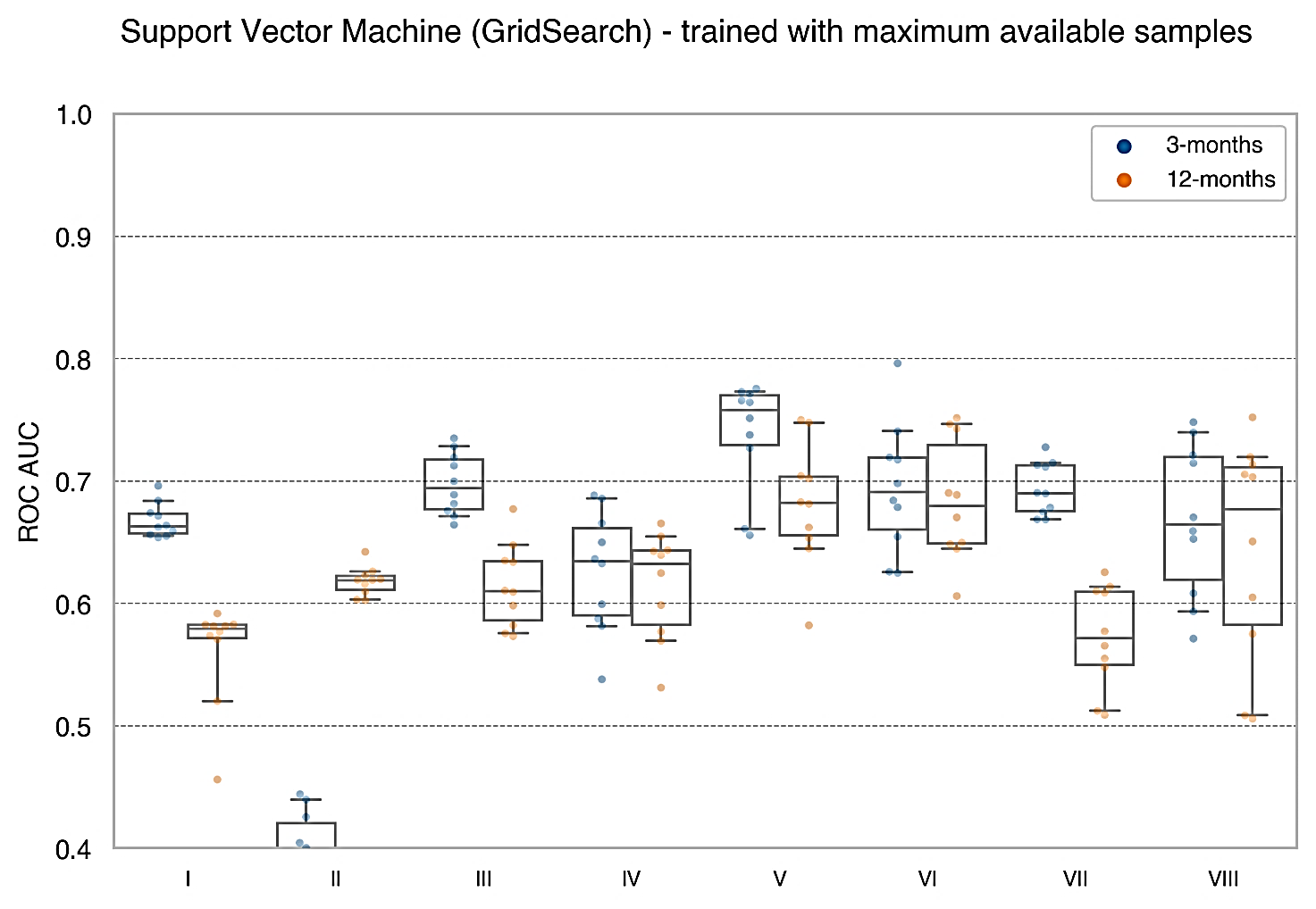


**Figure S2. Support Vector Machine EEG models with same sample data predicting 3- and 12-month outcomes.** Boxplots illustrating model performances (AUCs) of machine-learning models based on EEG-features predicting 3-month (**blue**) and 12-month (**orange**) functional outcomes. Each model is based on the same samples (n=58) for head-to-head comparison of EEG-features. Of the unimodal models (**Ia-IIIa**), model **Ia** (i.e., Synek model) and **IIIa** (i.e., P(MCS) model) outperformed model **IIa** (i.e., ABCD model) in predicting 3-month outcome (AUC_Synek_ 0.70 [0.68-0.73] vs. AUC_P(MCS)_ 0.72 [0.68-0.78] vs. AUC_ABCD_ 0.43 [0.35-0.53]). In predicting 12-month outcome, model **IIa** (i.e., ABCD model) and **IIIa** (i.e., P(MCS) model) outperformed model **Ia** (i.e., Synek model) (AUC_ABCD_ 0.67 [0.65-0.69] vs. AUC_P(MCS)_ 0.63 [0.50-0.71] vs. AUC_Synek_ 0.47 [0.28-0.58]). Of the combined models, models IVa-VIa and VIIIa could predict 3- and 12-month outcomes, and none outperformed the others. Model Viia could not predict 12-month outcome. *Individual same-sample EEG SVM-models:* **Ia**=Synek, **IIa**=ABCD, **IIIa**=P(MCS) C. *Combined same-sample EEG SVM-models*: **IVa**=Synek + ABCD, **Va**=Synek + ABCD + EEG markers-r, **VIa**= Synek + ABCD + P(MCS), **VIIa**= Synek + P(MCS) and **VIIa**= Synek + ABCD + P(MCS) + EEG markers-r


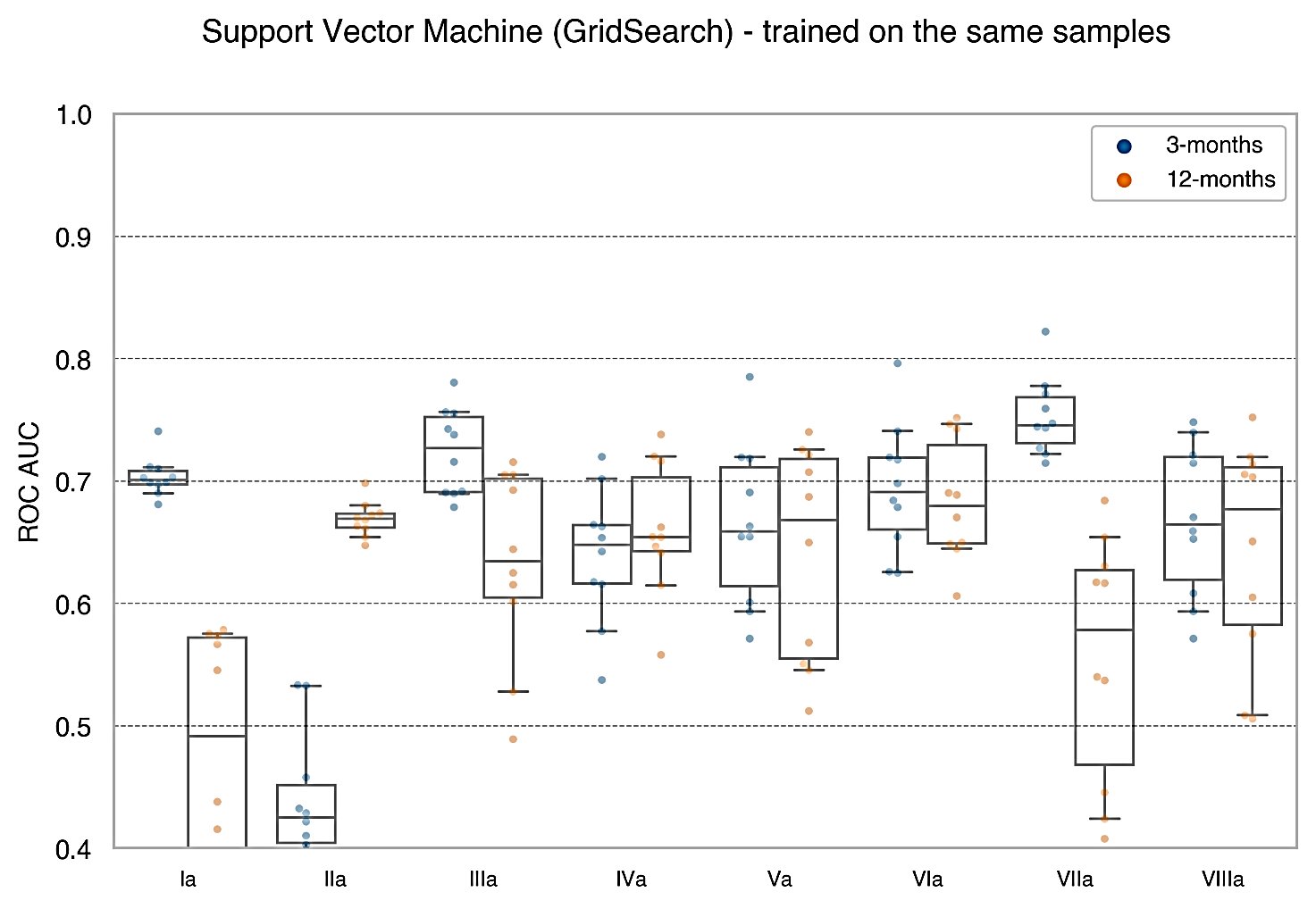


**Figure S3. SHAP analysis of EEG features included in EEG model VIII.** SHAP analysis of the EEG model including all EEG features (model VIII), using SHAP (Shapley additive explanation). This SHAP analysis summarizes the impact of each feature in the model’s output by ranking all 68 EEG markers-r as well as the Synek score, ABCD characterization and P(MCS). The features with highest rank (overall power spectral density, beta power spectral density and P(MCS) derived from EEG resting segments) are placed on top of the figure.


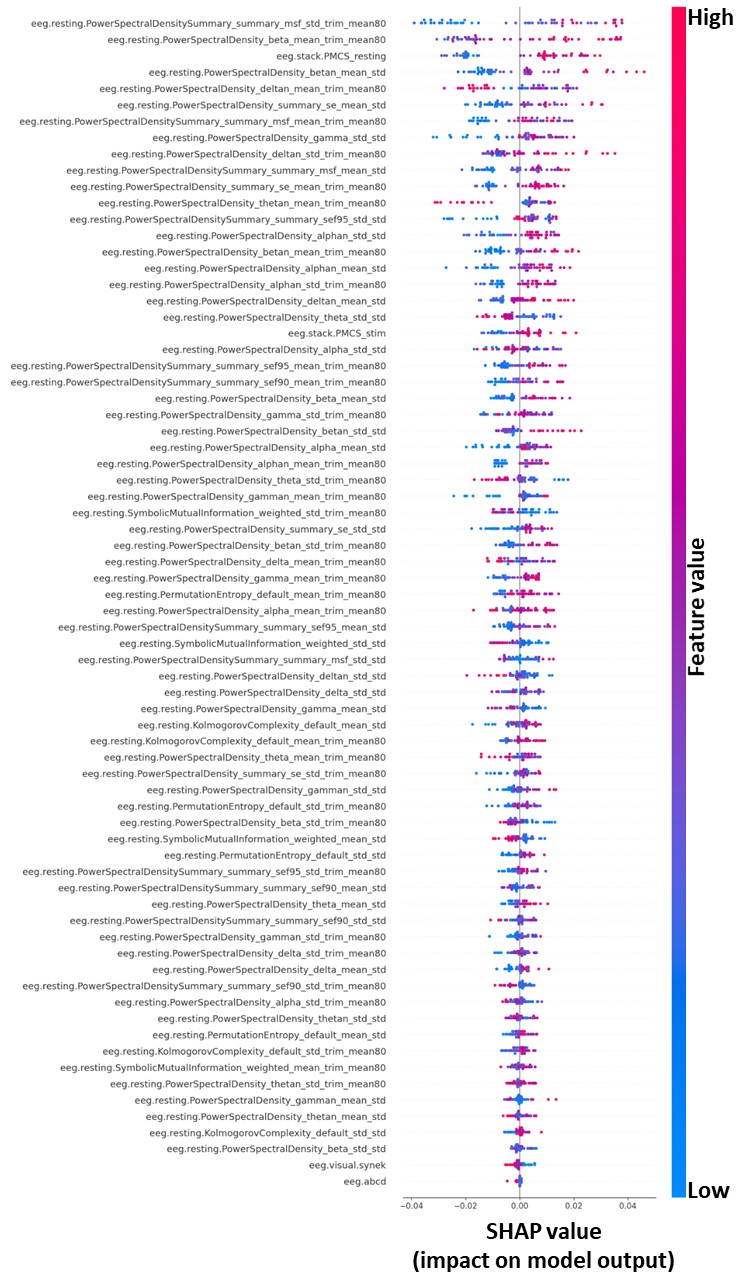

Supplement: Supplementary file 1 — Supplementary file1 (DOCX 828 KB) [file 12028_2023_1816_MOESM1_ESM.docx]
